# Impact of Small Body Weight on Tenofovir-Associated Renal Dysfunction in HIV-Infected Patients: A Retrospective Cohort Study of Japanese Patients

**DOI:** 10.1371/journal.pone.0022661

**Published:** 2011-07-25

**Authors:** Takeshi Nishijima, Hirokazu Komatsu, Hiroyuki Gatanaga, Takahiro Aoki, Koji Watanabe, Ei Kinai, Haruhito Honda, Junko Tanuma, Hirohisa Yazaki, Kunihisa Tsukada, Miwako Honda, Katsuji Teruya, Yoshimi Kikuchi, Shinichi Oka

**Affiliations:** 1 AIDS Clinical Center, National Center for Global Health and Medicine, Tokyo, Japan; 2 Department of Community Care, Saku Central Hospital, Nagano, Japan; 3 Center for AIDS Research, Kumamoto University, Kumamoto, Japan; University of Cape Town, South Africa

## Abstract

**Background:**

Treatment with tenofovir is sometimes associated with renal dysfunction. Limited information is available on this side effect in patients with small body weight, although the use of tenofovir will spread rapidly in Asia and Africa, where patients are likely to be of smaller body weight.

**Methods:**

In a single-center cohort, Japanese patients with HIV infection who started tenofovir-containing antiretroviral therapy were retrospectively analyzed. The incidence of tenofovir-associated renal dysfunction, defined as more than 25% decrement of estimated glomerular filtration rate (eGFR) from the baseline, was determined. The effects of small body weight and body mass index (BMI) on tenofovir-associated renal dysfunction, respectively, were estimated in univariate and multivariate Cox hazards models as the primary exposure. Other possible risk factors were evaluated by univariate analysis and those found significant were entered into the multivariate analysis.

**Results:**

The median weight of 495 patients was 63 kg. Tenofovir-related renal dysfunction occurred in 97 (19.6%) patients (incidence: 10.5 per 100 person-years). Univariate analysis showed that the incidence of tenofovir-related renal dysfunction was significantly associated with smaller body weight and BMI, respectively (per 5 kg decrement, HR = 1.23; 95% CI, 1.10–1.37; p<0.001)(per 1 kg/m^2^ decrement, HR = 1.14; 95% CI, 1.05–1.23; p = 0.001). Old age, high baseline eGFR, low serum creatinine, low CD4 count, high HIV viral load, concurrent nephrotoxic drugs, hepatitis C infection, and current smoking were also associated with tenofovir-related renal dysfunction. Multivariate analysis identified small body weight as a significant risk (adjusted HR = 1.13; 95% CI, 1.01–1.27; p = 0.039), while small BMI had marginal significance (adjusted HR = 1.07; 95% CI 1.00–1.16; p = 0.058).

**Conclusion:**

The incidence of tenofovir-associated renal dysfunction in Japanese patients was high. Small body weight was identified as an independent risk factor for tenofovir-associated renal dysfunction. Close monitoring of renal function is advocated for patients with small body weight treated with tenofovir.

## Introduction

Tenofovir disoproxil fumarate (TDF) is one of the most widely used nucleotide reverse transcriptase inhibitors (NRTI) for patients with HIV infection, with proven efficacy and safety [1]–[6]. However, TDF is known to cause renal proximal tubular dysfunction, and several case reports have been published with TDF-related Fanconi syndrome, diabetes insipidus, and acute tubular necrosis, which sometimes lead to acute renal failure [7]–[10]. Long-term TDF use also reduces glomerular filtration rate more than other NRTIs [11]–[14]. To date, the nephrotoxic effect of TDF is regarded as mild and tolerable. A recently published meta-analysis has reported that the use of TDF is associated with a statistically significant but only modest renal dysfunction, and recommended that TDF use should not be restricted even when regular monitoring of renal function and serum phosphate levels is impractical [15]. However, the TDF-related renal dysfunction has hardly been evaluated in patients with small body weight, who are potentially at higher risk for larger drug exposure and thus, more severe toxicity [16]–[19].

The 2010 WHO guideline on antiretroviral therapy for HIV infection in adults and adolescents, usually applied to resource-constrained settings, recommends TDF as one of the components of first line therapies (URL:http://whqlibdoc.who.int/publications/2010/9789241599764_eng.pdf). It is expected that the use of TDF will spread rapidly in Asia and Africa in the near future, where patients are more likely to be of small body weight. Thus, at this stage, it is important to establish the relationship between TDF-associated renal dysfunction and body weight. A small body weight is considered a risk factor for TDF-associated renal dysfunction, in addition to old age, high baseline serum creatinine level, low CD4 count, concurrent use of ritonavir-boosted protease inhibitor, and concurrent use of nephrotoxic drugs [4], [17], [19]–[21]. To our knowledge, there is almost no report that primarily analyzed the influence of body weight on TDF-associated renal dysfunction. Since Japanese are generally of smaller stature and have a lower median body weight than Whites and African Americans, who mostly comprise the cohorts of studies published to date, it is important to investigate the impact of TDF-associated renal dysfunction in Japanese patients.

Based on the above background, the present study was designed to determine the incidence of TDF-associated renal dysfunction in Japanese patients and analyze the impact of small body weight on TDF-associated renal dysfunction.

## Methods

### Ethics Statement

This study was approved by the Human Research Ethics Committee of National Center for Global Health and Medicine (Text S1). All patients included in this study provided a written informed consent for their clinical and laboratory data to be used and be published for research purposes. This study has been conducted according to the principles expressed in the Declaration of Helsinki.

### Study Design and Settings

We performed a single-center, retrospective cohort study of HIV-infected Japanese patients using medical records at the National Center for Global Health and Medicine, Tokyo, Japan. Our facility is one of the largest clinics for patients with HIV infection in Japan with more than 2,700 registered patients.

### Study Subjects

The study population were patients >17 years of age who commenced treatment with standard 300 mg/day of TDF-containing antiretroviral regimen at our clinic between January 1, 2002 to March 31, 2009. Both treatment-naive and patients with experience in antiretroviral treatment but not TDF, with an estimated glomerular filtration rate (eGFR) of >60 ml/min/1.73 m^2^ were enrolled. Patients were followed up until September 31, 2009. Patients were excluded if their follow-up period at our facility was less than 24 weeks after commencement of TDF-based therapy, if they had started TDF at other facilities, or if there was evidence of prior TDF use. We only included Japanese patients in order to examine a population with comparatively homogenous basic demographics and background.

### Measurements

#### Outcome measure: TDF-associated renal dysfunction

We defined TDF-associated renal dysfunction as more than 25% decrease in eGFR relative to the baseline [17], [22], [23]. Baseline eGFR was estimated for each patient from the average of two successive serum creatinine measurements made closest to and preceding the commencement of TDF by no more than 90 days. Changes in eGFR were plotted from the baseline measurement until the value diminished to less than 75% of the baseline or at the end of the follow-up period. The eGFR values at occurrence of TDF-associated renal dysfunction, at censoring, and closest to and preceding 24, 48, and 96 weeks to the diagnosis were collected. Patients generally visited our clinic between every month to every 3 months, and measurement of eGFR was usually conducted on every visit. eGFR was calculated using the equation from the 4-variable Modification of Diet in Renal Disease (MDRD) study [24].

#### Primary exposure variable

Our primary exposure variables were body weight and body mass index (BMI) at the time of commencement of TDF-containing antiretroviral therapy (ART). BMI was calculated by the equation: BMI = [body weight (kg)/height (m)^2^].

#### Other variables: potential risk factors

Potential risk factors for TDF-associated renal dysfunction were determined according to previous studies and collected together with the basic demographics from the medical charts [4], [19], [20], [25]. They included sex, age, baseline laboratory data: CD4 cell count, HIV viral load, and serum creatinine, and other medical conditions (antiretroviral treatment-naïve or experienced, concurrent ritonavir-boosted protease inhibitors, concurrent nephrotoxic drugs such as ganciclovir, sulfamethoxazole/trimethoprim, ciprofloxacin, and NSAIDs, diabetes mellitus, co-infection with hepatitis B defined by positive hepatitis B surface antigen, co-infection with hepatitis C defined by positive HCV viral load, hypertension defined by current treatment with antihypertensive agents, dyslipidemia defined by current treatment with lipid-lowering agents, and current smoking) [26]. We used the data on or closest to and preceding the day of starting TDF-containing ART by no more than 90 days. The data on weight change from the baseline to the end of follow-up period and the frequency of eGFR monitoring for each patient were collected.

### Statistical analysis

The time to 25% decline in eGFR from the baseline was calculated from the date of treatment initiation to the date of occurrence of TDF-associated renal dysfunction. Censored cases represented those who discontinued TDF, dropped out, referred to other facilities, or at the end of follow-up period. The time from TDF initiation to 25% decrease in eGFR was analyzed by the Kaplan Meier method for the whole cohort. To estimate the impact of body weight on the incidence of TDF-associated renal dysfunction, we calculated the impact of every 5 kg decrement from the median weight using Cox proportional hazards regression analysis. The impact of every 1 kg/m^2^ decrement in BMI on the incidence of TDF-associated renal dysfunction was estimated by the same method. The impact of each basic demographics, baseline laboratory data, and other medical conditions listed above was also estimated with univariate Cox proportional hazards regression.

To estimate the unbiased prognostic impact of weight on TDF-associated renal dysfunction, we conducted three models using multivariate Cox proportional hazards regression analysis. Model 1 was the aforementioned univariate analysis for every 5 kg decrement. Model 2 included sex, age plus model 1 in order to adjust for basic characteristics. In model 3, we added variables with P values<0.05 in univariate analysis for adjustment (these included age per 10 years, serum creatinine >0.8 mg/dl, CD4 count <200/µl, HIV viral load per log10/ml, concurrent nephrotoxic drugs, co-infection with hepatitis C, and current smoking). Concurrent ritonavir-boosted protease inhibitors were also added in Model 3 although their p value was 0.116 in the univariate analysis. This was based on the results of several studies suggesting that concurrent use of ritonavir-boosted protease inhibitors is a risk factor for TDF-associated renal dysfunction [19], [20]. The eGFR was excluded from multivariate analysis because of its multicollinearity with sex, age, and serum creatinine, since eGFR was gained by the equation of those variables. The impact of every 1 kg/m^2^ decrement in BMI on the incidence of TDF-associated renal dysfunction was estimated by the same method with Model 1 to Model 3.

Four other analyses were conducted to further examine the relationship between low body weight and TDF-associated renal dysfunction. First, the time from initiation of TDF therapy to 25% decrease in eGFR was analyzed by the Kaplan Meier method for intertertile baseline body weight categories: <59, 59–67, and >67 kg. The log-rank test was used to determine statistical significance. Second, to investigate the impact of changes in muscle mass on changes in the eGFR as calculated by MDRD, we compared weight changes with one-way ANOVA among intertertile baseline weight categories. We also conducted the sensitivity analysis by adding the variable “weight change” in multivariate analysis. Third, the median and interquartile value for the actual fall in eGFR from the baseline to 24, 48, and 96 weeks for the whole cohort and three baseline weight categories, respectively, were calculated. The eGFR value at 24, 48, and 96 weeks included those that were censored before reaching 24, 48, and 96 weeks, respectively, so that we could interpret the data for actual fall in eGFR, including not only survived cases but also censored cases. Fourth, we counted the number of patients whose eGFR decreased to <60 and <10 ml/min/1.73 m^2^, and who discontinued TDF with the clinical diagnosis of renal dysfunction due to TDF. Chi-square test was used to determine whether the difference among the weight categories was statistically significant.

Statistical significance was defined at two-sided p values<0.05. We used hazard ratios (HRs) and 95% confidence intervals (CIs) to estimate the impact of each variable on TDF-associated renal dysfunction. All statistical analyses were performed with The Statistical Package for Social Sciences ver. 17.0 (SPSS, Chicago, IL).

## Results

**Figure 1:**
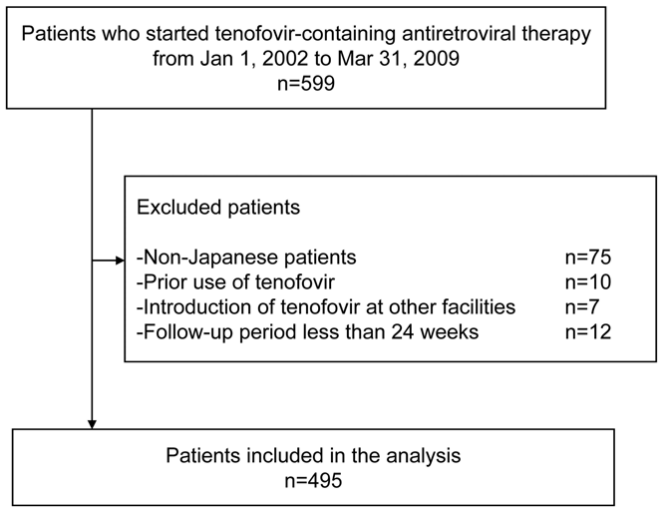
Flow diagram of patient selection.

Between January 1, 2002 to March 31, 2009, 599 patients started TDF-containing ART (Figure 1). Of these, 104 patients were excluded based on the abovementioned criteria. Thus 495 patients were included in the present study (Dataset S1). Table 1 shows the demographics, laboratory data, and medical conditions of the study population at baseline. Two patients received ART with 3 NRTIs, 3 patients received ART with one protease inhibitor (PI), one non-NRTI (NNRTI), and tenofovir/emtricitabine, and the remaining patients had a standard ART with 2 NRTIs and either PI, NNRTI, or integrase inhibitor (INI). The median body weight and BMI were 63 kg and 21.9 kg/m^2^, respectively. The median age of the patients was 38 years and 95.2% were males. The eGFR was well maintained (median: 120.9 ml/min/1.73 m^2^), and the median baseline CD4 count was 247/µl. Of the total, 208 patients (42%) were antiretroviral treatment naïve, while 287 were treatment-experienced patients. Viral load was suppressed to <50 copies/ml in 162 (32.7%) patients. 403 (81.4%) were on concurrent PIs as the key drug, 367 (74.1%) were on ritonavir-boosted PIs, and only 83 (16.8%) had NNRTIs as the key drug. Smoking was prevalent among the study population, as 240 (48.5%) were identified as a current smoker.

**Table 1:** Baseline demographics and laboratory data.

**Characteristics**		
Median (IQR) weight (kg)	63	(57–69)
Median (IQR) BMI (kg/m^2^)	21.9	(20.3–23.8)
Male, n (%)	471	(95.2)
Median (IQR) age	38	(33–46)
Median (IQR) eGFR (ml/min/1.73 m^2^)	120.9	(104.8–138.2)
Median (IQR) serum creatinine (mg/dl)	0.72	(0.64–0.81)
Median (IQR) CD4 count (/µl)	247	(159–371)
Median (IQR) HIV viral load (log10/ml)	3.73	(1.60–4.81)
HIV viral load <50 copies/ml, n (%)	162	(32.7)
Antiretroviral therapy naïve, n (%)	208	(42.0)
Key drugs, n (%)*		
PIs	403	(81.4)
Ritonavir-boosted PIs	367	(74.1)
LPV/r	175	(35.4)
ATV/r	131	(26.5)
FPV/r	52	(10.5)
DRV/r	9	(1.8)
FPV	14	(2.8)
ATV	4	(0.8)
NFV	15	(3)
SQV	2	(0.4)
IDV	1	(0.2)
NNRTIs	83	(16.8)
EFV	65	(13.1)
NVP	17	(3.4)
ETR	1	(0.2)
INI		
RAL	10	(2.0)
Concurrent use of nephrotoxic drug, n (%)	131	(26.5)
Diabetes mellitus, n (%)	30	(6.1)
Hepatitis B, n (%)	75	(15.2)
Hepatitis C, n (%)	52	(10.5)
Hypertension, n (%)	28	(5.7)
Dyslipidemia, n (%)	40	(8.1)
Smoking, n (%)	240	(48.5)
Median (IQR) weight change (kg)	0.0	(−2.0–2.25)
Median (IQR) frequency of eGFR monitoring	16	(9.0–27)

(n = 495).

* Two patients did not take any key drugs. Three patients took both PI and NNRTI.

IQR: interquartile range, BMI: body mass index, eGFR: estimated glomerular filtration rate, PI: protease inhibitor, LPV/r: lopinavir/ritonavir, ATV: atazanavir, FPV: fosamprenavir, DRV: darunavir, NFV: nelfinavir, SQV: saquinavir, IDV: indinavir, NNRTI: non-nucleos(t)ide reverse transcriptase inhibitor, EFV: efavirenz, NVP: nevirapine, ETR: etravirine, INI: integrase inhibitor, RAL: raltegravir.

**Figure 2:**
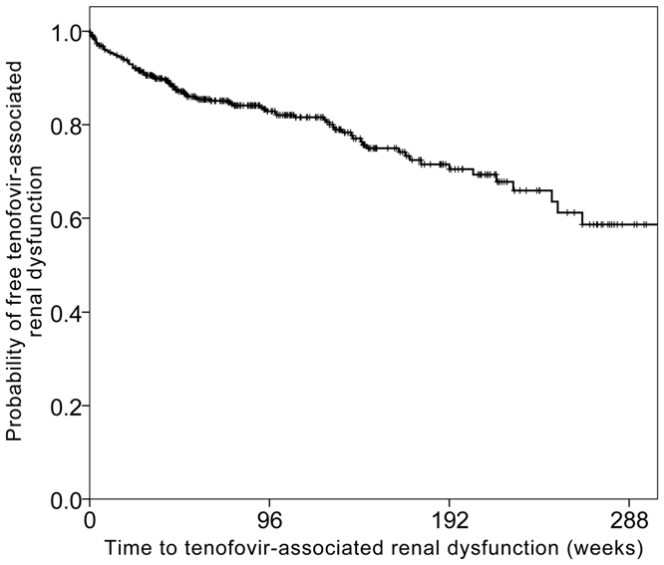
Kaplan-Meier curve showing the time to 25% reduction in eGFR for the whole cohort. eGFR: estimated glomerular filtration rate.

TDF-associated renal dysfunction defined by more than 25% decrease of eGFR from baseline occurred in 97 patients (19.6%), with an estimated incidence of 10.5 per 100 person-years. The median time from commencement of TDF to occurrence of TDF-associated renal dysfunction was 39 weeks (IQR 13.5–99.4 weeks) (range: 1–1,841 days). The total observation period was 924.7 patient-years (median 72 weeks, IQR 38.6–139.3 weeks). Figure 2 shows the Kaplan-Meier survival curve for the occurrence of TDF-associated renal dysfunction for the whole cohort.

Univariate analysis showed a significant relationship between TDF-associated renal dysfunction and every 5 kg less than the median body weight (HR = 1.23; 95% CI, 1.10–1.37; p<0.001), and 1 kg/m^2^ less BMI than the median BMI (HR = 1.14; 95% CI, 1.05–1.23; p = 0.001) (Table 2). Furthermore, old age, high eGFR, low serum creatinine, low CD4 counts, high HIV viral load, concurrent use of nephrotoxic drugs, presence of chronic hepatitis C, and smoking were associated with TDF-related renal dysfunction. On the other hand, concurrent use of PIs, ritonavir boosted PIs, and LPV/r tended to be associated with TDF-related renal dysfunction, albeit statistically insignificant. Treatment-naïve or Treatment-experienced was not associated with TDF-related renal dysfunction.

**Table 2:** Univariate analysis for TDF-associated renal dysfunction.

	HR	95%CI	P value
Weight per 5 kg decrement	1.23	1.10–1.37	<0.001
BMI per 1 kg/m^2^ decrement	1.14	1.05–1.23	0.001
Male gender	0.54	0.26–1.11	0.094
Age per 10 years	1.22	1.02–1.45	0.027
eGFR per 10 ml/min/1.73 m^2^	1.10	1.05–1.15	<0.001
Serum creatinine >0.8 mg/dl	0.51	0.30–0.88	0.014
CD4 count <200/µl	1.97	1.32–2.93	0.001
HIV viral load per log10/ml	1.15	1.01–1.30	0.037
Antiretroviral therapy naïve	0.98	0.63–1.52	0.927
Concurrent key drugs			
Any PIs	1.52	0.89–2.59	0.124
Ritonavir boosted PIs	1.46	0.91–2.33	0.116
LPV/r	1.45	0.97–2.17	0.072
ATV/r	1.05	0.66–1.68	0.826
Concurrent nephrotoxic drug	1.59	1.04–2.42	0.031
Diabetes mellitus	1.57	0.76–3.24	0.220
Hepatitis B	1.36	0.82–2.24	0.231
Hepatitis C	1.80	1.07–3.04	0.028
Hypertension	1.18	0.51–2.69	0.702
Dyslipidemia	0.97	0.47–2.00	0.932
Smoking	1.57	1.05–2.36	0.028

TDF: tenofovir, HR: hazard ratio, CI: confidence interval, BMI: body mass index, eGFR: estimated glomerular filtration rate, PI: protease inhibitor, LPV/r: lopinavir/ritonavir, ATV: atazanavir.

Multivariate analysis showed that every 5 kg less than the median body weight was a significant risk for TDF-associated renal dysfunction after adjustment for sex and age (adjusted HR = 1.21; 95% CI, 1.07–1.36; p = 0.002) (Table 3, Model 2), and also after adjustment for other risk factors (adjusted HR = 1.13; 95% CI, 1.01–1.27; p = 0.039) (Table 3, Model 3). Similarly, every 1 kg/m^2^ less than the median BMI was also a significant risk factor for TDF-associated renal dysfunction even after adjustment for sex and age (adjusted HR = 1.13; 95% CI 1.05–1.22; p = 0.002) (Table 4, Model 2), and tended to be a significant factor after adjustment for other variables (adjusted HR = 1.07; 95% CI 1.00–1.16; p = 0.058) (Table 4, Model 3). Old age and current smoking were also independent risk factors in both multivariate analysis for body weight and BMI (Table 3, Model 3 and Table 4, Model 3).

**Table 3:** Multivariate analysis to estimate the effect of lower body weight on TDF-associated renal dysfunction.

	Model 1 Crude		Model 2 Adjusted		Model 3 Adjusted	
	HR	95%CI	HR	95%CI	HR	95%CI
Weight per 5 kg decrement¶	1.23	1.10–1.37	1.21	1.07–1.36	1.13	1.01–1.27
Male gender			0.88	0.41–1.89	0.57	0.26–1.26
Age per 10 years¶			1.16	0.98–1.38	1.24	1.04–1.49
Serum creatinine >0.8 mg/dl					0.62	0.35–1.07
CD4 count <200/µl					1.65	0.97–2.79
HIV viral load per log10/ml					1.05	0.90–1.23
Boosted PIs					1.54	0.93–2.54
Concurrent use of nephrotoxic drug					1.23	0.77–1.97
Hepatitis C					1.57	0.92–2.69
Smoking¶					1.65	1.09–2.48

¶ P<0.05 in Model 3.

TDF: tenofovir, HR: hazard ratio, CI: confidence interval, PI: protease inhibitor.

**Table 4:** Multivariate analysis to estimate the impact of BMI decrement on TDF-associated renal dysfunction.

	Model 1 Crude		Model 2 Adjusted		Model 3 Adjusted	
	HR	95%CI	HR	95%CI	HR	95%CI
BMI per 1 kg/m^2^ decrement	1.14	1.05–1.23	1.13	1.05–1.22	1.07	1.00–1.16
Male gender			0.67	0.32–1.38	0.48	0.23–1.03
Age per 10 years¶			1.20	1.01–1.43	1.27	1.06–1.52
Serum creatinine >0.8 mg/dl					0.60	0.35–1.04
CD4 count <200/µl					1.64	0.97–2.79
HIV viral load per log10/ml					1.05	0.90–1.23
Boosted PIs					1.49	0.90–2.45
Concurrent use of nephrotoxic drugs					1.22	0.76–1.94
Hepatitis C					1.62	0.94–2.76
Smoking¶					1.63	1.08–2.46

¶ P<0.05 in Model 3.

BMI: body mass index, TDF: tenofovir, HR: hazard ratio, CI: confidence interval, PI: protease inhibitor.

**Figure 3:**
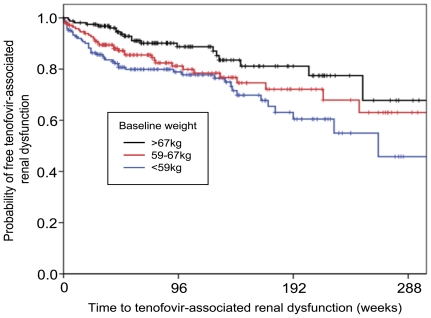
Kaplan-Meier curve showing the time to 25% reduction in eGFR according to baseline weight categories. Compared to patients with body weight >67 kg, those with weight <59 kg were more likely to develop >25% decline in eGFR (P = 0.002), whereas those with weight 59–67 kg showed only a marginal significance (P = 0.073, log-rank test). eGFR: estimated glomerular filtration rate.

In complementary analyses, First, Figure 3 shows the relation between probability of TDF-associated nephrotoxicity and time from initiation of TDF therapy to 25% decrease in eGFR analyzed by the Kaplan Meier method for intertertile baseline weight categories. Compared to patients with baseline body weight >67 kg, patients with baseline weight <59 kg were significantly more likely to develop >25% decline in eGFR (p = 0.002). On the other hand, the difference in this probability between patients with baseline weight 59–67 kg and those >67 kg was only marginally significant (p = 0.073, log-rank test). Secondly, one-way ANOVA showed that weight changes among the three baseline weight categories were not significantly different (p = 0.206). Sensitivity analysis after adding the variable “weight change” in Model 3 multivariate analysis (Table 3) showed that adjusted hazard ratio for weight per 5 kg decrement hardly changed (adjusted HR 1.131; 95% CI, 1.007–1.271; p = 0.038). Thirdly, Table 5 shows the median and interquartile values for the actual falls in eGFR from the baseline to 24, 48, and 96 weeks. The eGFR decreased gradually in all categories, except for patients with baseline weight >67 kg. Fourthly, the number (percentage) of patients whose eGFR decreased to <60 ml/min/1.73 m^2^ was not different among the baseline weight categories (p = 0.229), whereas the number of patients who discontinued TDF with a clinical diagnosis of renal dysfunction due to TDF varied significantly according to body weight (p = 0.001, chi-square test, Table 6). None of the patients showed reduction of eGFR to <10 ml/min/1.73 m^2^.

**Table 5:** Median and interquartile range of the actual fall in eGFR from the baseline to 24, 48, and 96 weeks, according to body weight.

	Total (n = 495)	<59 kg (n = 167)	59–67 kg (n = 168)	>67 kg (n = 160)
	fall in eGFR(ml/min/1.73 m^2^)		fall in eGFR		fall in eGFR		fall in eGFR	
	median	IQR	median	IQR	median	IQR	median	IQR
to 24 weeks	7.8	(−1.7–18.1)	9.8	(−3.6–22.6)	6.8	(−1.5–17.3)	7.3	(−1.8–15.4)
to 48 weeks	9.0	(−0.7–21.9)	13.0	(−0.2–29.3)	7.2	(−1.2–20.0)	8.1	(−0.6–18.6)
to 96 weeks	9.3	(−0.5–23.1)	13.4	(1.2–33.2)	8.6	(−0.2–21.7)	7.5	(−2.4–19.8)

eGFR: estimated glomerular filtration rate, IQR: interquartile range.

**Table 6:** Number of patients whose eGFR decreased to <60 ml/min/1.73 m^2^ and who discontinued tenofovir with clinical diagnosis of renal dysfunction due to tenofovir.

	<59 kg (n = 167)	59–67 kg (n = 168)	>67 kg (n = 160)	p value
eGFR <60 ml/min/1.73 m^2^	4 (2.4%)	1 (0.6%)	1 (0.6%)	0.229
Discontinued tenofovir	16 (9.6%)	8 (4.8%)	1 (0.6%)	0.001
Reasons for discontinuation				
>25% eGFR decrement	8 (4.8%)	4 (2.4%)	0 (0%)	
Urine β2 microglobulin >5000 µg/l	11 (6.6%)	4 (2.4%)	1 (0.6%)	

Among the patients who discontinued tenofovir, both >25% fall in eGFR and urine β2 microglobulin >5000 µg/l were registered in six patients with body weight <59 kg, and in three patients with body weight 59–67 kg.

eGFR: estimated glomerular filtration rate.

## Discussion

In this Japanese cohort, 19.6% of the patients experienced eGFR decline of more than 25% from the baseline after commencement of TDF. The incidence of TDF-associated renal dysfunction was 10.5 per 100 person-years. Multivariate analysis identified smaller body weight and smaller body mass index as significant and almost significant factors, respectively, for TDF-associated renal dysfunction.

The incidence of TDF-associated renal dysfunction in patients with small body weight might be higher than previously reported in studies of patients with larger statures. Studies from North America, Europe, and Australia reported an incidence of <1% to 4.3% for TDF-related renal dysfunction, although the definition used for the diagnosis of renal impairment was different among the studies and varied from an increase in serum creatinine from >0.5 to >2 mg/dL from baseline [1]–[3], [5], [13]. Several studies conducted in these regions indicated that the range of patients' mean body weight was 69–74 kg, indicating that their patients were heavier than those of the present study with a median weight of 63 kg [2], [6], [12], [14]. The impact of the comparatively lower body weight seems stronger in our patients probably because they do not appear to have many of the other established risk factors for TDF-associated renal dysfunction despite the high incidence of 10.5 per 100 person-years. For example, they were comparatively young with a median age of 38 years, CD4 count was relatively maintained, and approximately 30% had suppressed HIV viral load at baseline (Table 1). Furthermore, they were less likely to have hypertension, dyslipidemia, and diabetes mellitus.

The results of multivariate analysis that each 5 kg decrement in body weight was significantly associated with TDF-associated renal dysfunction but not each 1 kg/m^2^ decrement in BMI suggests that weight might be more useful and handy information to estimate the risk for TDF-associated renal dysfunction than BMI. Thus, patient's body weight is an important risk factor to consider at the time of TDF prescription.

Our study is one of a few that have examined the impact of TDF-associated renal dysfunction in patients with small body weight, but is the first to examine the impact of small body weight as a primary exposure by creating the model used for multivariate analysis [16]–[18]. One study from Thailand that included patients with a median weight of 56.5 kg reported a similar incidence of 16.2 per 100 person-years for developing TDF-associated renal dysfunction [17]. They concluded that the small body weight of their patients was probably associated with the high incidence of TDF-associated renal dysfunction. Our study confirmed that conclusion and provided statistically-backed evidence that small body weight is a significant risk factor of TDF-associated renal dysfunction by using a multivariate model with least multicollinearity to evaluate the impact of small body weight. The results of the present study could be applied to many countries in Asia and Africa, where stature and body weight of the population are comparatively smaller.

This study adopted a decrease in eGFR of >25% as a definition for TDF-associated renal dysfunction. This criterion is one of common methods in evaluating renal function [22], [23]. Using this definition, however, does not mean that all patients with >25% fall in eGFR have severe renal dysfunction. However, the definition of renal dysfunction based on a fall in eGFR of >25% is probably more sensitive than that based on eGFR <60 ml/min/1.73 m^2^, in patients with comparatively good baseline renal function, such as patients of our study. Adopting this definition could be useful in detecting early renal dysfunction and in the clinical decision making regarding the need for certain interventions, for example, discontinuation of TDF. Early detection of renal dysfunction is particularly important in patients with HIV infection, because kidney disease may be associated with AIDS and death, and TDF-associated renal dysfunction might be irreversible [27], [28].

Since the calculation of eGFR using the MDRD formula is based on serum creatinine, age, race and gender, any fall in eGFR is influenced by hypercreatininemia caused by increased muscle mass [29]. It is possible that the muscle mass increases in patients on ART, especially those with low weight at baseline compared to those with higher weight, reflecting reversal of wasting in those patients who were most malnourished. Such increase in muscle mass could then result in a fall in eGFR despite no change in actual renal elimination of creatinine. However, complementary analysis showed that weight change throughout the follow-up period was not significantly different among patients with different baseline weight, and the sensitivity analysis demonstrated that weight change did not alter the significance of every 5 kg decrement.

In the present study, high eGFR and low serum creatinine levels at baseline were identified as risk factors for falls in eGFR of more than 25%, in contrast to several previous studies that showed high serum creatinine and low eGFR were risk factors [4], [10], [25]. While the exact reason for this discrepancy is unknown at present, it could be related to differences in the definition of TDF-associated renal dysfunction. The aforementioned Thai study used the same definition applied in the present study and a Canadian study that used the definition of 1.5-fold increase in serum creatinine from baseline also reported high eGFR and low serum creatinine level at baseline as risk factors [17], [30]. Thus, it is plausible to observe a fall in eGFR when the baseline value is high, since Horberg et al. reported that patients with baseline eGFR of >80 ml/min/1.73 m^2^ were likely to show a pronounced fall in eGFR with TDF use [31].

Multivariate analysis also suggested that old age and current smoking are significant risks for TDF-associated renal dysfunction (Table 3, Model 3 and Table 4, Model 3). However, these results have to be interpreted with caution, because these multivariate analyses were formulated to primarily evaluate weight decrement, not age or smoking.

The mechanism of TDF-associated renal dysfunction is not fully understood. TDF-associated renal dysfunction probably develops as a result of complex interaction of pharmacological, environmental, and genetic factors, rather than small body weight only [32]. It should be noted, however, that small body weight has been identified as a risk factor for TDF-associated renal dysfunction not only in clinical trials, but also in *in vitro* and pharmacokinetic studies [33]–[36]. TDF is the prodrug of acyclic nucleotide analog tenofovir, which is excreted by both glomerular filtration and active tubular secretion. *In vitro* studies showed that tenofovir exhibits mitochondrial toxicity in renal proximal tubular cells, and animal studies demonstrated that renal tubular dysfunction was associated with the dose and plasma drug concentrations of TDF [34], [35]. Furthermore, pharmacokinetic studies showed that small body weight is associated with reduced plasma TDF clearance and thus high plasma TDF concentrations, which could result in renal tubular dysfunction. [33], [36].

There are several limitations to our study. First, because of the retrospective nature of the study, patients with possible risks for TDF-associated renal dysfunction could have not been prescribed TDF. Because of this selection bias, the incidence of TDF-associated renal dysfunction might be underestimated. Second, the study did not compare the incidence of renal dysfunction in a control group (TDF-free ART). Due to the small body weight in Japanese or other factors such as genetics, the use of ART without TDF might cause higher incidence of renal dysfunction as well. Third, as discussed above, the definition of TDF-associated renal dysfunction, especially the criteria used to evaluate proximal renal tubular damage, is not uniformly established in the field and is different in the published studies. Accordingly, we decided to adopt changes in eGFR, instead of parameters for proximal renal tubular damage. Using the eGFR as a marker for TDF-associated renal dysfunction, our results might have underestimated the incidence of TDF-associated renal dysfunction.

In conclusion, the present study demonstrated a high incidence of TDF-associated renal dysfunction among Japanese patients, a potentially high-risk group due to the low median body weight. The results also identified small body weight as a risk for TDF-associated renal dysfunction in a statistical model that included small body weight as a primary exposure. TDF is certainly a drug of choice for one of the components of the first line therapies for HIV infection. However, the importance of close monitoring for renal function in patients with small body weight should be emphasized for early detection of TDF-associated renal dysfunction.

## Supporting Information

Text S1 **Letter of Approval from Human Research Ethics Committee of National Center for Global Health and Medicine.**
(PDF)

Dataset S1 **Raw data of the target population.**
(XLS)
