# Supplementary material for: Impact of Small Body Weight on Tenofovir-Associated Renal Dysfunction in HIV-Infected Patients: A Retrospective Cohort Study of Japanese Patients
Source: PLoS One. 2011 Jul 25;6(7):e22661. doi: 10.1371/journal.pone.0022661 (PMC3143186; doi:10.1371/journal.pone.0022661)
Supplement: Text S1 — Letter of Approval from Human Research Ethics Committee of National Center for Global Health and Medicine. (PDF) [file pone.0022661.s001.pdf]

独立行政法人
国立国際医療研究センター
NCGM National Center for Global Health and Medicine

Letter of Approval
Human Research Ethics Committee

November 4th, 2010

Takeshi Nishijima, M.D.
AIDS Clinical Center
National Center for Global Health and Medicine

Dear Dr. Nishijima,

Re: Impact of Tenofovir-associated Renal Dysfunction among HIV-infected Patients with Low Body-weight: A Retrospective Cohort Study of the Japanese patients

Corresponding researcher: Hiroyuki Gatanaga, MD., PhD.

Your correspondence and attachments were noted by the National Center for Global Health and Medicine Research Ethics Committee at its meeting on October 28th, 2010. The committee reviewed the study protocol, which were approved. Please quote the following reference number on any future correspondence with the committee regarding the protocol: 920

Sincerely,

Takaaki Kirino, M.D., PhD.
President
National Center for Global Health and Medicine
